# Book Reviews

**Published:** 1913-02

**Authors:** 


### BOOK REVIEWS

PERSONAL HYGIENE AND PHYSICAL TRAINING FOR WOMEN. By Anna M. Galbrath, M. D., Fellow of the New York Academy of Medicine; author of "The Four Epochs of Woman's Life." 12 mo. of 371 pages, illustrated. Cloth $2.00 net. W. B. Saunders & Co., Philadelphia.

This book contains much needed information.

---

CLINICAL DIAGNOSIS: A Manual of Laboratory Methods. By James Campbell Todd, M. D., Professor of Pathology, University of Colorado. 12mo of 469 pages, with 164 text-illustrations and 11 colored plates. Cloth, $2.25 net. W. B. Saunders & Co., Philadelphia.

The new matter added to this edition includes bacteriologic methods, preparation and use of vaccines and tuberculins, the antiformin method for tubercle bacilli, detection of albumin in sputum, Tsuchiya's modification of Esbach's test, formation test for ammonia in urine, Wright and Kinnicutt method of counting blood-platelets, Harlow's blood-stain and the Wassermann reaction.

---

DYSPEPSIA. By William Soltau Fenwick, M. D., of London, England. Octavo volume of 485 pages, illustrated. Cloth, $3.00 net. W. B. Saunders & Co., Philadelphia.

Fenwick discusses in a thorough and systematic way the causes, pathology, symptoms, diagnosis prognosis and treatment of these diseases in a definite concise manner, which at once appeals to the reader on account of its practical value.

NERVOUS AND MENTAL DISEASES. By Archibald Church, M. D., Professor of Nervous and Mental Diseases and Medical Jurisprudence, Northwestern University Medical School, Chicago; and Frederick Peterson, M. D., Professor of Psychiatry, College of Physicians and Surgens, New York. Handsome octavo of 930 pages, with 341 practical illustrations. Cloth, $5.00 net; Half Morocco, $6.50 net. W. B. Saunders & Co., Philadelphia.

This seventh edition has been thoroughly revised. The chapters on Meningitis, Aphasia, Poliomyelitis, Pellagra, and Pituitary Diseases have been practically rewritten. A chapter on Oppenheim's Congenital Amyatonia has been introduced. The section on Mental Diseases has been wholly rearranged to conform to the latest classification, some obsolete matter struck out, and much new matter added.

---

A COLLECTION OF PAPERS (Published Previous to 1909.) By William J. Mayo, M.D., and Charles H. Mayo, M.D., Two octavo volumes, averaging 550 pages each, illustrated. Philadelphia and London: W. B. Saunders Company, 1912. Per set, cloth, $10.00 net.

This volume has been prepared to preserve a complete file of the writings of W. J. and C. H. Mayo, from the time of their graduation to February 1909, the date of the publication, the first volume of collected papers by the staff of St. Mary's Hospital. The work of the Mayo's is too well known to need comment. Their writings reflect the advance guard of surgical progress.

---

DISEASES OF THE SKIN AND THE ERUPTIVE FEVERS. By Jay F. Schamberg, M. D., Professor of Dermatology and the Infectious Eruptive Diseases, Philadelphia Polyclinic. Octavo of 573 pages, illustrated. Cloth, $3.00 net. Second edition. W. B. Saunders & Co., Philadelphia.

This is an excellent up-to-date book which reflects especially modern work in actinotherapy, Rontgenotherapy and radium.

HYDROTHERAPY: A Treatise on Hydrotherapy in General; Its Application to Special Affections; the Technic or Process Employed, and a Brief Chapter on the Use of Waters Internally. By Guy Hinsdale, M. D., Fellow of the Royal Society of Great Britain. Octavo of 466 pages, illustrated. Cloth $3.50 net. W. B. Saunders & Co., Philadelphia.

This book describes fully the various kinds of baths, douches, sprays; the application of heat and cold; the internal use of mineral waters and all other procedures included under hydrotherapeutic measures.

---

THE PRINCIPLES AND PRACTICE OF PHYSICAL DIAGNOSIS. By John C. DaCosta, Jr., M. D., Assistant Professor of Clinical Medicine, Jefferson Medical College. Octavo of 557 pages, with 212 original illustrations. Cloth, $3.50 net. W. B. Saunders & Co., Philadelphia.

Dr. DaCosta's work is a thoroughly new and original one. Every method given has been carefully tested and proved of value by the author himself. Normal physical signs are explained in detail in order to aid the diagnostician in determining the abnormal; and the pathologic significance of abnormal physical signs is clearly indicated. Both direct and differential diagnosis are emphasized.

---

A MANUAL OF PRACTICAL HYGIENE. For Students, Physicians and Health Officers, by Charles Harrington, M.D., late Professor of Hygiene in the Medical School of Harvard University. Fourth edition, revised and enlarged by Mark W. Richardson, M. D., Secretatry to State Board of Health of Massachusetts. Octavo, 850 pages, with 124 engravings and 12 full-page plates, in colors and monochrome. Cloth, $4.50 net. Lea & Febiger, Philadelphia and New York, 1911.

The medical profession may advisably take note of the growing public interest in all matters pertaining to the maintenance of health, an indication that the physician is now expected to be quite an expert preventing disease as in curing it. In fact, the intelligent portion of the laity is becoming exceedingly well informed on matters of Hygiene, and it behooves the profession to lead instead of being pushed along its own ground. Fortunately is has at hand in the new edition of Harrington's Hygiene a standard authority, thoroughly revised to date, complete in every department and detail and easy and delightful to read.

---

PRACTICAL TREATMENT. By 82 eminent specialists. Edited by John H. Musser, M. D., Professor of Clinical Medicine, and A. O. J. Kelly, M. D., late Assistant Professor of Medicine, University of Pennsylvania. Three octavos of 950 pages each, illustrated. Per volume: Cloth, $6.00 net; Half Morocco, $7.50 net. Subscription. W. B. Saunders & Co., Philadelphia.

This is probably one of the best works upon this subject in English.

---

THE PATHOLOGY AND TREATMENT OF SEXUAL IMPOTENCE. By Victor G. Vecki, M. D., Consulting Genito-Urinary Surgeon to the Mount Zion Hospital, San Francisco. 12 mo of 400 pages. W. B. Saunders & Co., Philadelphia.

This new edition reflects all the recent work in urology. The chapters on Anatomy and Physiology have been greatly enlarged, but by far the most important and extensive changes are in the chapters on treatment which afford the best guide we have in the management of this disorder.

---

INFANT FEEDING. By Clifford G. Grulee, A.M., M.D., Assistant Professor of Pediatrics at Rush Medical College, Attending Pediatrician to Cook County Hospital. Octavo of 295 pages, illustrated. Philadelphia and London: W. B. Saunders Company, 1812. Cloth, $3.00 net.

Grulee has collected the knowledge of the processes which underlie infant feeding up to the present and has applied this knowledge in a clear and practical manner so that the information may be grasped by one no more familiar with the subject than the practicing physician.

SURGICAL AFTER-TREATMENT. By L. R. G. Crandon, M.D., Assistant in Surgery at Harvard Medical School, and Albert Ehrenfried, M.D., Assistant in Anatomy at Harvard Medical School. Second edition, practically re-written. Octavo of 831 pages, with 264 original illustrations. Philadelphia and London: W. B. Saunders Company, 1912. Cloth, $6.00 net; Half Morocco, $7.50 net.

This splendid and exceedingly useful work has been revised, as the shift of medical opinion and the advance of medical knowledge have required, many changes have therefore been made and some chapters entirely re-written.

---

DIGESTION AND METABOLISM. The Physiological and Pathological Chemistry of Nutrition, by Alonzo E. Taylor, Rush Professor of Physiological Chemistry, University of Pennsylvania, Philadelphia. Lea & Febiger, Phila. and New York, 1912.

This work presents the closely related subjects of digestion and metabolism in a thorough and scholarly manner. The author endeavors to describe the chemical changes in normal and abnormal digestion, and explain the known metabolic modifications that food materials undergo in the body.

A clear understanding of these subjects greatly aids in the comprehension of the pathology of diseases termed metabolic—such as gout, diabetes, nephritis, and the legion of autointoxications.

This is a carefully prepared and well written book, and will repay the reader for painstaking study. A cursory survey, however, will not yield much knowledge; nor can it be recommended as "light summr reading."

According to St. Paul's classificatiton of "milk for babes and meat for strong men", this work decidedly comes under the second heading.

G. M. N.

A TREATISE ON DISEASES OF THE HAIR. By George Thomas Jackson, M.D., Professor of Dermatology in the University of Columbia, and Charles Wood McMurtry,

M.D., Instructor in Dermatology in the College of Physicians and Surgeons, Medical Department of Columbia University, New York. Octavo, 366 pages, with 109 engravings and 10 colored plates. Cloth, $3.75, net. Lea & Febiger, Philadelphia and New York, 1912.

In recognition of the need for an adequate presentation of this important subject, Drs. Jackson and McMurtry have created the first work in this field which may justly claim to be authoritative, scientific and thoroughly practical. The sections on diagnosis and treatment have been made full and precise, and have been prefaced with a discussion of the pathology of the disease under consideration. The latest researches are included. An especially attractive feature has been made of the illustrations and colored plates.

